# Comparison of Childless and Partnerless Vasectomy Rates Before and After *Dobbs v. Jackson Women’s Health Organization*

**DOI:** 10.1177/15579883241260511

**Published:** 2024-06-13

**Authors:** Vi Nguyen, Michelle K. Li, Michelle C. Leach, Darshan P. Patel, Tung-Chin Hsieh

**Affiliations:** 1 Department of Urology, UC San Diego Health, University of California, San Diego, La Jolla, CA, USA

**Keywords:** vasectomy, reproductive health, reproductive legislation

## Abstract

The Supreme Court ruling *Dobbs v. Jackson Women’s Health Organization* (June 2022) overturned federal protection of abortion rights, resulting in significant impact on both male and female reproductive rights and health care delivery. We conducted a retrospective review of all patients who underwent vasectomy at a single academic institution between June 2021 and June 2023. Our objective was to compare the rates of childless and partnerless vasectomies 1 year before and after this ruling, as these men may be more susceptible to postprocedural regret. Of total, 631 men (median age = 39 years, range = 20–70) underwent vasectomy consultation. Total vasectomies pre- and post-Dobbs were 304 (48%) versus 327 (52%). Total childless and partnerless vasectomies pre- and post-Dobbs were 44 (42%) versus 61 (58%) and 43 (46%) versus 50 (54%). Vasectomy completion rate was slightly increased post-Dobbs (90% vs. 88%; *p* = .240). The post-Dobbs cohort had significantly less children (1.8 vs. 2.0; *p* = .031). Men in the post-Dobbs era were significantly more likely to be commercially insured (72% vs. 64%) and less likely to be uninsured (1% vs. 6%; *p* = .002). Men who underwent childless vasectomy were significantly more likely to be younger (36.4 vs. 39.8 years; *p* < .001). There was a significantly greater proportion of Hispanic and Black men in the partnerless cohort compared to the cohort with partners (24% vs. 19% and 9% vs. 2%; *p* = .002). In conclusion, patients should be counseled on the permanent nature of this procedure, underscoring need for effective and reversible male contraception.

## Introduction

On June 24, 2022, the United States Supreme Court issued its ruling in *Dobbs v. Jackson Women’s Health Organization*, stating that “the Constitution is neutral and leaves the issue for the people and their elected representatives to resolve through the democratic process in the States or Congress” (United States Reports, 2022). The passage effectively overturned *Roe et al. v. Wade, District Attorney of Dallas County* (1973), which established a constitutional right to abortion through the second trimester of pregnancy. Since the Dobbs ruling, 14 states have completely banned abortion (Choi & Cole, 2024). While media and research have largely been focused on the legislature’s implications on women’s reproductive rights, there are limited studies on the effects of this legal decision on men (Berg & Woods, 2023). Currently, contraceptive options for men are limited to abstinence, withdrawal prior to ejaculation, condoms, and vasectomy (Thirumalai & Page, 2020). Of these options, vasectomy remains the only reliable method (Thirumalai & Amory, 2021). Short-term studies have demonstrated rising vasectomy volume after the Dobbs ruling; in particular, with a 225% increase in the immediate 3 months following the decision (Bole et al., 2024; Zhang et al., 2023; Zhu et al., 2024).

We sought to compare vasectomy consultation and completion trends 1 year pre- and post-Dobbs specifically in childless and partnerless men, as this population may be more vulnerable to postprocedural regret (Anderson et al., 2022; Charles et al., 2023). Consequently, this may result in additional reversal procedures and health care costs (Pavlovich & Schlegel, 1997). These data may be helpful in providing patient-centered vasectomy consultations in the setting of changes in reproductive legislature and state-dependent policies.

## Methods

This was a retrospective review of all patients who underwent vasectomy within the Department of Urology at a single academic institution in San Diego, California, between June 2021 and June 2023 to capture a study period encompassing 1 year pre- and post-Dobbs.

All data were collected from the electronic medical record (EMR) and de-identified. Institutional review board (IRB) exemption was obtained. Demographic variables that were collected for analysis included age, race/ethnicity, body mass index (BMI), insurance coverage (commercial vs. government vs. uninsured), number of children at time of vasectomy (i.e., childless status), and partner status at time of vasectomy. Periprocedural data collected include date of vasectomy consultation, date of vasectomy procedure, duration between consultation and procedure (months), vasectomy completion, vasectomy setting (clinic vs. operating room), and prior history of vasectomy, vasovasostomy, or epididymovasostomy.

Of note, race/ethnicity was self-reported by patients in the EMR; thus, this information was missing for some patients as some declined to report. Government insurance included Medicare, Medicaid/Medi-Cal, and military/Tricare coverage. It is important to note that the average cost for vasectomy among patients who self-pay ranges from $1,429.74 to $3,185.37 (Mortach et al., 2024). Number of children at time of vasectomy referred to biological children. Partnership included both marriage and a relationship. This information was recorded from the clinic note during vasectomy consultation, as these questions are standardly asked by all our providers for this type of encounter.

All patients who underwent vasectomy prior to July 2022 were stratified into the pre-Dobbs cohort, whereas patients who underwent vasectomy from July 2022 and onward were stratified into the post-Dobbs cohort.

The primary outcome of the study was comparison of number of vasectomies among childless and partnerless men pre- versus post-Dobbs. Secondary outcomes included investigating differences between these populations in regard to demographic variables.

All statistical analysis was conducted utilizing SPSS version 28. Descriptive statistics were performed. The bivariate Pearson correlation test was used to determine correlation between continuous variables. The chi-square test of independence was used to determine correlation between categorical variables. The one-way analysis of variance (ANOVA) was used to assess correlation between continuous and categorical variables.

## Results

A total of 631 men were seen for vasectomy consultation during the study period. Median age at time of vasectomy was 39 years (range = 20–70). Median BMI was 27.1 kg/m^2^ (range = 18.4–54.7). Racial distribution was as follows: 59% White (*n* = 370/631), 20% Hispanic (*n* = 125/631), 7% Asian (*n* = 47/631), 3% Black (*n* = 18/631), and 11% Other/Unreported (*n* = 71/631). Insurance coverage of the cohort was as follows: 69% commercial (*n* = 432/631), 28% government (*n* = 179/631), and 3% without insurance (*n* = 20/631).

Median time to vasectomy from initial consultation was 1 month (range = 1–23). 89% (*n* = 559/631) of men who were seen for vasectomy went through with the procedure. 99% (*n* = 551/559) of vasectomies were performed in clinic rather than the operating room. No patients had a prior history of previous vasectomy, vasovasostomy, or epididymovasostomy. Median number of children prior to vasectomy was 2 (range = 0–6). 17% (*n* = 105/631) of men were childless at time of vasectomy. 15% (*n* = 93/631) of men were partnerless at time of vasectomy. The total number of men who were both childless and partnerless was *n* = 73/631 (12%).

**Figure 1.**
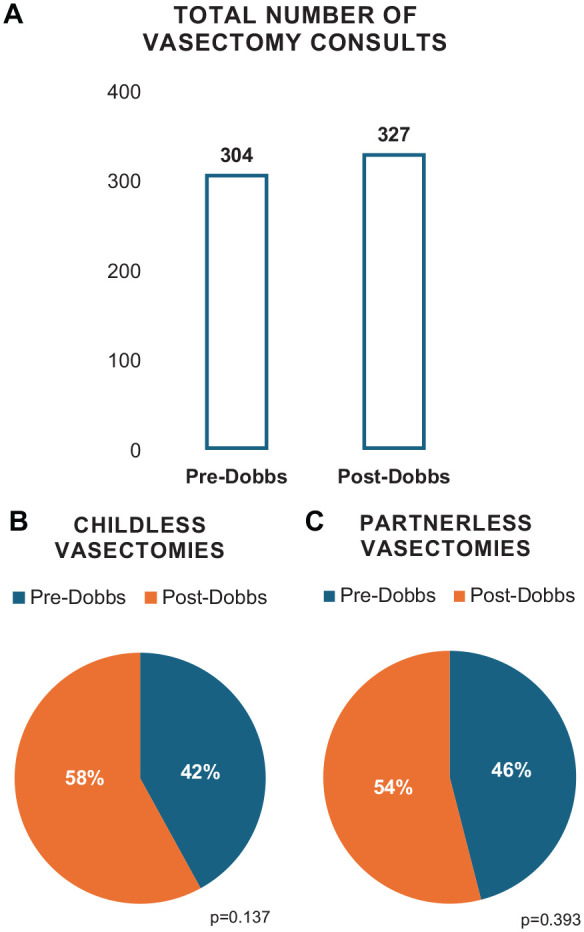
Total Number of (A) Vasectomy Consultations, (B) Childless Vasectomies, and (C) Partnerless Vasectomies One Year Pre- and Post-Dobbs

Total number of vasectomies pre- and post-Dobbs were 304 (48%) and 327 (52%), respectively (Figure 1A). Total number of vasectomies in childless men pre- and post-Dobbs was 44 (42%) and 61 (58%; Figure 1B), and total number of vasectomies in partnerless men pre- and post-Dobbs was 43 (46%) and 50 (54%; Figure 1C).

There was a greater proportion of vasectomies among childless men in the post-Dobbs time period (19% vs. 14%; *p* = .137), although this did not reach statistical significance. However, patients in the post-Dobbs cohort had a significantly lower number of children (1.8 vs. 2.0; *p* = .022). There was also a slight increase in vasectomy completion rate in the post-Dobbs era (90% vs. 88%; *p* = .240), although not statistically significant. Men in the post-Dobbs era were significantly more likely to be commercially insured (72% vs. 64%) and less likely to be uninsured (1% vs. 6%; *p* = .002; Table 1).

**Table 1.** Comparison of Pre-Dobbs and Post-Dobbs Vasectomy Cohort

Demographic variable	Total cohort (*n* = 631)	Pre-Dobbs^a^ cohort (*n* = 304)	Post-Dobbs^b^ cohort (*n* = 327)	*p* ^c^
*M*±*SD* Age (Years)	39.2 ± 6.9	39.2 ± 6.8	39.2 ± 6.9	.992
*M*±*SD* BMI (kg/m^2^)	27.9 ± 4.7	28.1 ± 4.7	27.7 ± 4.7	.361
Race				
*White*	59% (*n* = 370)	59% (*n* = 177)	58% (*n* = 193)	.480
*Hispanic*	20% (*n* = 125)	20% (*n* = 60)	20% (*n* = 65)	
*Asian*	7% (*n* = 47)	9% (*n* = 28)	6% (*n* = 19)	
*Black*	3% (*n* = 18)	3% (*n* = 9)	3% (*n* = 9)	
*Other*	11% (*n* = 71)	10% (*n* = 30)	12% (*n* = 41)	
Insurance Coverage				
*Commercial*	69% (*n* = 432)	64% (*n* = 196)	72% (*n* = 236)	**.002**
*Government*	28% (*n* = 179)	30% (*n* = 91)	27% (*n* = 88)	
*Uninsured*	3% (*n* = 20)	6% (*n* = 17)	1% (*n* = 3)	
*M*±*SD* Number of Children	1.9 ± 1.2	2.0 ± 1.2	1.8 ± 1.1	**.022**
Childless (%)	17% (*n* = 105)	14% (*n* = 44)	19% (*n* = 61)	.137
Partnerless (%)	15% (*n* = 93)	14% (*n* = 43)	15% (*n* = 50)	.393
Vasectomy Completion Rate (%)	89% (*n* = 559)	88% (*n* = 266)	90% (*n* = 293)	.240
M ±*SD* Time to Vasectomy (months)	2.0 ± 2.1	1.9 ± 2.0	2.1 ± 2.1	.442
In Clinic Vasectomy (%)	99% (*n* = 551)	99% (*n* = 263)	98% (*n* = 288)	.417

Bold values represent significant *p*-value <0.05.

a Pre-Dobbs: prior to July 2022. ^b^Post-Dobbs: July 2022 onwards. ^c^ *p*: chi-square test of independence for categorical variables, bivariate Pearson correlation test for continuous variables.

Among the total cohort, childless men who underwent vasectomy were significantly more likely to be younger (36.4 vs. 39.8 years; *p* < .001) and have a lower BMI (26.8 vs. 28.1 kg/m^2^; *p* = .009; Table 2). Within the partnerless cohort, Hispanic (24% vs. 19%) and Black men (9% vs. 2%; *p* = .002; Table 2) comprised a larger proportion compared to the cohort with partners. Younger age remained a significant predictor of vasectomy among childless men in the pre-Dobbs cohort (36.5 vs. 39.7 years; *p* = .004; Table 3) and post-Dobbs cohort (36.4 vs. 39.9 years; *p* < .001; Table 4). Black men comprised a larger proportion of the partnerless cohort post-Dobbs (10% vs. 1%; *p* = .004; Table 4).

**Table 2.** Characteristics of Childless and Partnerless Vasectomy Cohort

Demographic variable	Childless cohort (*n* = 105)	With child cohort (*n* = 526)	*p* ^a^	Partnerless cohort (*n* = 93)	With partner cohort (*n* = 538)	*p* ^a^
*M*±*SD* Age (Years)	36.4 ± 8.3	39.8 ± 6.4	**<.001**	39.2 ± 6.5	39.5 ± 9.0	.733
*M*±*SD* BMI (kg/m^2^)	26.8 ± 4.7	28.1 ± 4.7	**.009**	27.7 ± 5.3	27.9 ± 4.7	.757
Race						
*White*	65% (*n* = 68)	57% (*n* = 302)	.717	57% (*n* = 53)	59% (*n* = 317)	**.002**
*Hispanic*	17% (*n* = 18)	20% (*n* = 107)		24% (*n* = 22)	19% (*n* = 103)	
*Asian*	6% (*n* = 6)	8% (*n* = 41)		5% (*n* = 5)	8% (*n* = 42)	
*Black*	3% (*n* = 3)	3% (*n* = 15)		9% (*n* = 8)	2% (*n* = 10)	
*Other*	9% (*n* = 10)	12% (*n* = 61)		5% (*n* = 5)	12% (*n* = 66)	
Insurance Coverage						
*Commercial*	67% (*n* = 70)	69% (*n* = 362)	.863	66% (*n* = 61)	69% (*n* = 371)	.274
*Government*	30% (*n* = 32)	28% (*n* = 147)		33% (*n* = 31)	27% (*n* = 148)	
*Uninsured*	3% (*n* = 3)	3% (*n* = 17)		1% (*n* = 3)	4% (*n* = 17)	
Vasectomy Completion Rate (%)	88% (*n* = 92)	89% (*n* = 467)	.420	88% (*n* = 82)	89% (*n* = 477)	.503

Bold values represent significant *p*-value <0.05.

a *p*: chi-square test of independence for categorical variables, bivariate Pearson correlation test for continuous variables.

**Table 3.** Characteristics of Childless and Partnerless Vasectomy Cohort Pre-Dobbs

Demographic variable	Childless cohort (*n* = 44)	With child cohort (*n* = 260)	*p* ^a^	Partnerless cohort (*n* = 43)	With partner cohort (*n* = 261)	*p* ^a^
*M*±*SD* Age (Years)	36.5 ± 9.2	39.7 ± 6.3	**.004**	40.7 ± 9.5	39.0 ± 6.3	.122
*M*±*SD* BMI (kg/m^2^)	27.3 ± 5.5	28.2 ± 4.6	.246	27.9 ± 5.7	28.1 ± 4.6	.800
Race						
*White*	59% (*n* = 26)	58% (*n* = 151)	.918	53% (*n* = 23)	59% (*n* = 154)	.344
*Hispanic*	20% (*n* = 9)	20% (*n* = 51)		26% (*n* = 11)	19% (*n* = 49)	
*Asian*	9% (*n* = 4)	9% (*n* = 24)		7% (*n* = 3)	10% (*n* = 25)	
*Black*	5% (*n* = 2)	3% (*n* = 7)		7% (*n* = 3)	2% (*n* = 6)	
*Other*	7% (*n* = 3)	10% (*n* = 27)		7% (*n* = 3)	10% (*n* = 27)	
Insurance Coverage						
*Commercial*	59% (*n* = 26)	65% (*n* = 170)	.717	65% (*n* = 28)	64% (*n* = 168)	.584
*Government*	34% (*n* = 15)	30% (*n* = 76)		33% (*n* = 14)	30% (*n* = 77)	
*Uninsured*	7% (*n* = 3)	5% (*n* = 14)		2% (*n* = 1)	6% (*n* = 16)	
Vasectomy Completion Rate (%)	91% (*n* = 40)	87% (*n* = 226)	.460	86% (*n* = 37)	88% (*n* = 229)	.756

Bold values represent significant *p*-value <0.05.Pre-Dobbs: prior to July 2022.

a *p*: chi-square test of independence for categorical variables, bivariate Pearson correlation test for continuous variables.

**Table 4.** Characteristics of Childless and Partnerless Vasectomy Cohort Post-Dobbs

Demographic variable	Childless cohort (*n* = 61)	With child cohort (*n* = 266)	*p* ^a^	Partnerless cohort (*n* = 50)	With partner cohort (*n* = 277)	*p* ^a^
*M*±*SD* Age (Years)	36.4 ± 7.7	39.9 ± 6.6	**<.001**	38.4 ± 8.6	39.4 ± 6.6	.336
*M*±*SD* BMI (kg/m^2^)	26.4 ± 4.1	28.0 ± 4.8	**.017**	27.6 ± 5.0	27.7 ± 4.7	.867
Race						
*White*	69% (*n* = 42)	57% (*n* = 151)	.485	60% (*n* = 30)	59% (*n* = 163)	**.004**
*Hispanic*	15% (*n* = 9)	21% (*n* = 56)		22% (*n* = 11)	20% (*n* = 54)	
*Asian*	3% (*n* = 2)	6% (*n* = 17)		4% (*n* = 2)	6% (*n* = 17)	
*Black*	2% (*n* = 1)	3% (*n* = 8)		10% (*n* = 5)	1% (*n* = 4)	
*Other*	11% (*n* = 7)	13% (*n* = 34)		4% (*n* = 2)	14% (*n* = 39)	
Insurance Coverage						
*Commercial*	72% (*n* = 44)	72% (*n* = 192)	.700	66% (*n* = 33)	73% (*n* = 203)	.376
*Government*	28% (*n* = 17)	27% (*n* = 71)		34% (*n* = 17)	26% (*n* = 71)	
*Uninsured*	0% (*n* = 0)	1% (*n* = 3)		0% (*n* = 0)	1% (*n* = 3)	
Vasectomy Completion Rate (%)	85% (*n* = 52)	91% (*n* = 241)	.216	90% (*n* = 45)	90% (*n* = 248)	.920

Bold values represent significant *p*-value <0.05.Post-Dobbs: July 2022 onwards.

a *p*: chi-square test of independence for categorical variables, bivariate Pearson correlation test for continuous variables.

## Discussion

A prior study from Michigan demonstrated that childless and partnerless men were 2.85- to 3.66-fold more likely to seek vasectomy post-Dobbs (Zhu et al., 2024). Herein, we sought to assess the incidence of vasectomy specifically within the childless and partnerless population in an extended 1-year study period prior to and after the *Dobbs v. Jackson Women’s Health Organization* Supreme Court ruling in 2022. Furthermore, we sought to examine if those findings could be replicated in a different geographic setting, as our academic center is based in San Diego, California, and our patient population may experience different accessibility compared to patients who reside in other regions.

Our findings demonstrate a greater number of vasectomy consultations in the post-Dobbs era. Though not statistically significant, we demonstrate a small increase in vasectomy completion rate post-Dobbs, indicating that perhaps the increased interest in vasectomy was matched with increased follow through.

Our findings indicate that not only were childless and partnerless men were more likely to seek vasectomy post-Dobbs, but also these childless men were also more likely to be younger. Studies have demonstrated that vasectomy regret rate is approximately 4%–6% among men who undergo this procedure (Anderson et al., 2022; Charles et al., 2023). Key social and demographic factors to be considered include age at time of procedure, parental status, and partner status. For example, men in their twenties were 12.5 times more likely to undergo vasectomy reversal compared to their older counterparts (95% confidence interval [CI]: [7.6, 20.7]) (Potts et al., 1999). Likewise, a separate study revealed that 94% of men who regretted their vasectomy had entered a new relationship following their procedure (Rungby et al., 1994). Not only are these procedures associated with their inherent surgical and anesthetic risks, they also incur significant health care system costs associated with vasectomy reversal ($24,475) and sperm retrieval with intracytoplasmic sperm injection ($72,521) (Pavlovich & Schlegel, 1997).

Future directions include further studies with longer follow-up duration to evaluate whether there is a corresponding increase in incidence of vasectomy reversal procedures concordant to the increased incidence of childless and partnerless vasectomies, which may indicate postprocedural regret. Male contraception options include abstinence, withdrawal prior to ejaculation, utilization of condoms, and vasectomy (Kogan & Wald, 2014). However, these options present with limitations. Condoms are associated with failure rates up to 13% with typical use (Sundaram et al., 2017). Despite the availability of reversal procedures, vasectomy is still regarded as a permanent sterilization procedure for men with fecundity anxiety. Thus, it is critical to develop safe, effective, and reversible male contraceptive options. Currently, there have been several studies investigating hormonally based male contraceptive regimens (Thirumalai & Page, 2020). Meanwhile, it is imperative to appropriately counsel all males, including childless and partnerless patients, regarding the risks, benefits, and alternatives of vasectomy prior to proceeding with the procedure.

There were several limitations to our study, the first being its retrospective nature. Given patients evaluated were from a single academic institution, findings may not be generalizable to patient populations from other practice settings or geographic location. There may be variance regarding vasectomy trends within the United States given geography is intimately linked to political climate and legislation. Our study was conducted in San Diego, California, where abortion rights are protected by the state constitution, covered by state Medicaid, and legal until fetal viability (gestational period of about 24–26 weeks) (Guttmacher Institute, 2024). Thus, it is critical to replicate this study in different regions of the United States where the legislative climate varies widely. Furthermore, our study population was predominantly White, which represents the demographics of the patient population at our medical center; thus, it remains unknown if cultural factors may impact decision-making regarding vasectomy as minority men were underrepresented in our cohort. Next, we were unable to ascertain if the partnerless population had partners prior or assess the sexuality of patients as this information was not consistently recorded in the medical record. We also did not gather any subjective data from the patients regarding the reason for electing for vasectomy; a future area of study would be to administer questionnaires to gain this perspective. In addition, we did not collect information regarding the completion rate of post-vasectomy semen analysis, which may reflect patients’ greater need to have confirmation that the procedure was successful. This is a variable that may be explored in future studies. Finally, we recognize that our study was limited by the short period of 1 year preceding and following the Supreme Court ruling. However, we elected to not capture data from a longer time period due to concern for confounding rates of access to care during the COVID-19 pandemic. Our department also experienced growth during the 2 to 3 years before the ruling; thus, the overall total number of vasectomies would have likely increased due to increased number of providers, which would subsequently confound our data.

## Conclusion

There was an increase in vasectomy consults, vasectomy completion rate, childless vasectomies, and partnerless vasectomies in the post-Dobbs study period. Younger men were more likely to undergo childless vasectomy. A greater proportion of Hispanic and Black men underwent partnerless vasectomy. Patients should be counseled on the permanent nature of this procedure, underscoring need for effective and reversible male contraception.
